# Supplementary material for: Microtubule minus-end aster organization is driven by processive HSET-tubulin clusters
Source: Nat Commun. 2018 Jul 9;9:2659. doi: 10.1038/s41467-018-04991-2 (PMC6037785; doi:10.1038/s41467-018-04991-2)
Supplement: Supplementary file 1 — Supplementary Information [file 41467_2018_4991_MOESM1_ESM.pdf]

Microtubule minus-end aster organization is driven by processive HSET-tubulin clusters

Norris et al. (2018)

Supplementary Figures 1-7

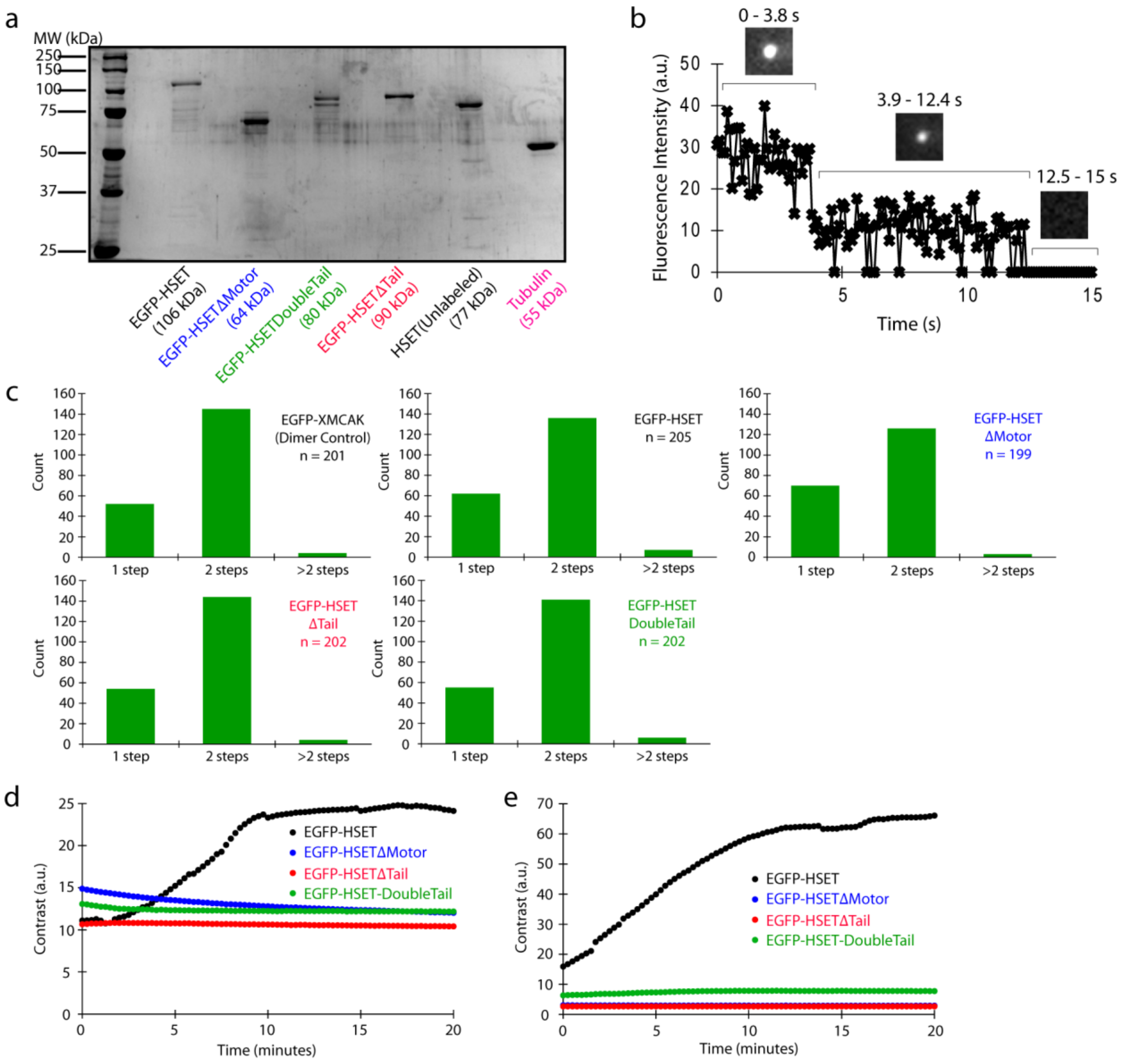

Supplementary Figure 1. Characterization of HSET constructs. (a) After two-step purification, 750 ng of the indicated construct was separated by SDS-PAGE and detected by Coomassie blue. MW markers are indicated on the left, and the predicted MW from the amino acid sequence for each construct is indicated in parentheses. **(b)** Representative two-step photobleaching of EGFP-HSET. EGFP-HSET was diluted to single-molecule concentrations, adhered to a glass surface, visualized by high-speed TIRF, and the Gaussian intensity was monitored over time. Insets depict averaged images over the indicated time period for the trace provided. **(c)** Quantification of stepwise photobleaching for each construct. The number of photobleaching steps was quantified for individual molecules and the population data were plotted as histograms, where the number of particles *n* from 2 independent experiments is indicated (N ≥ 4 movies for each condition). **(d)** Representative time course of self-organization (single fields corresponding to (Fig. 1b)) as monitored by contrast (standard deviation) in the tubulin channel. **(e)** Representative time course of self-organization (single fields corresponding to (Fig. 1c)) as monitored by contrast (standard deviation) in the tubulin channel.

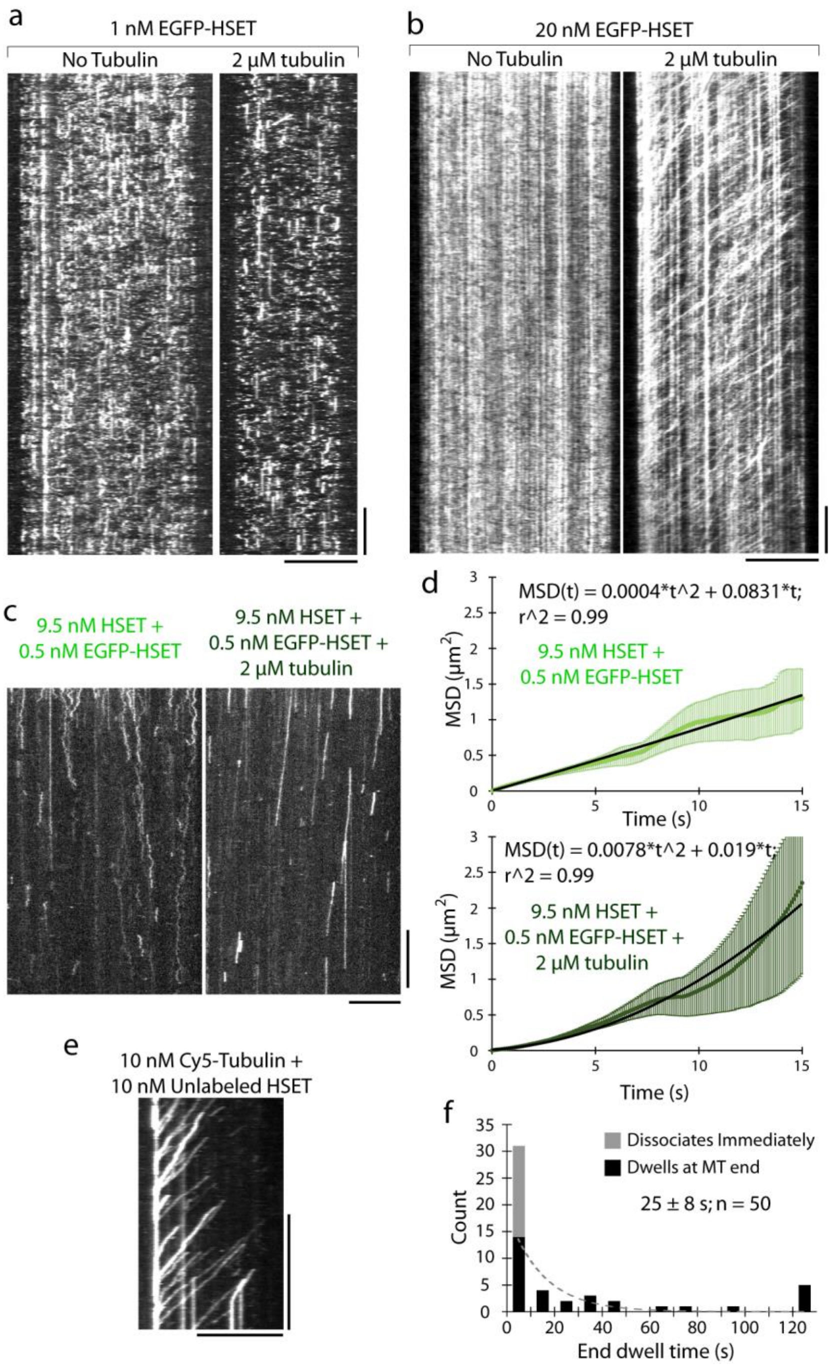

Supplementary Figure 2. Tubulin activates directed motility of HSET on single MTs. (a-b). 1 nM **(a)** or 20 nM **(b)** EGFP-HSET in BRB80 + 50 mM KCl was monitored by time-lapse TIRF in the absence (left) or presence (right) of soluble tubulin and visualized by kymograph. Distance is on the x-axis (scale bar, 10 µm), and time is on the y-axis (scale bar, 1 min). **(c)** 10 nM HSET (5% EGFP-labeled) in P12 buffer was monitored by high-speed time-lapse TIRF in the presence or absence of 2 µM tubulin and visualized by kymograph (x-scale bar, distance, 10 µm; y-scale bar, time, 10 s). **(d)** MSD quantification of **(**a). Data are presented as the calculated mean MSD (y-axis) from two independent experiments over the indicated time intervals (x-axis), ± SEM.

Solid lines indicate quadratic best fits for the indicated condition, where fits were obtained over the first five seconds with zero origin. Equations and corresponding r^2 values are shown as insets. n = 215 particles for 2 µM tubulin, n = 175 particles for no tubulin. Particles from 2 independent experiments are shown, where N ≥ 4 movies for each condition. **(e)** Representative kymograph of tubulin transport by HSET. Unlabeled HSET (10 nM) was mixed with Cy5-tubulin (10 nM) in BRB80 + 50 mM KCl, monitored by TIRF, and visualized by kymograph (x-scale bar, distance, 10 µm; y-scale bar, time, 10 s). **(f)** End dwell times for Cy5-tubulin were determined by kymograph analysis and plotted as histograms for the population. Data is reported as the mean values from CDF fitting, ± the 95% CI from bootstrapping from two independent experiments where N ≥ 4 movies for each condition. Particles dissociating immediately (<1 frame) are color-coded and were excluded from exponential CDF fits.

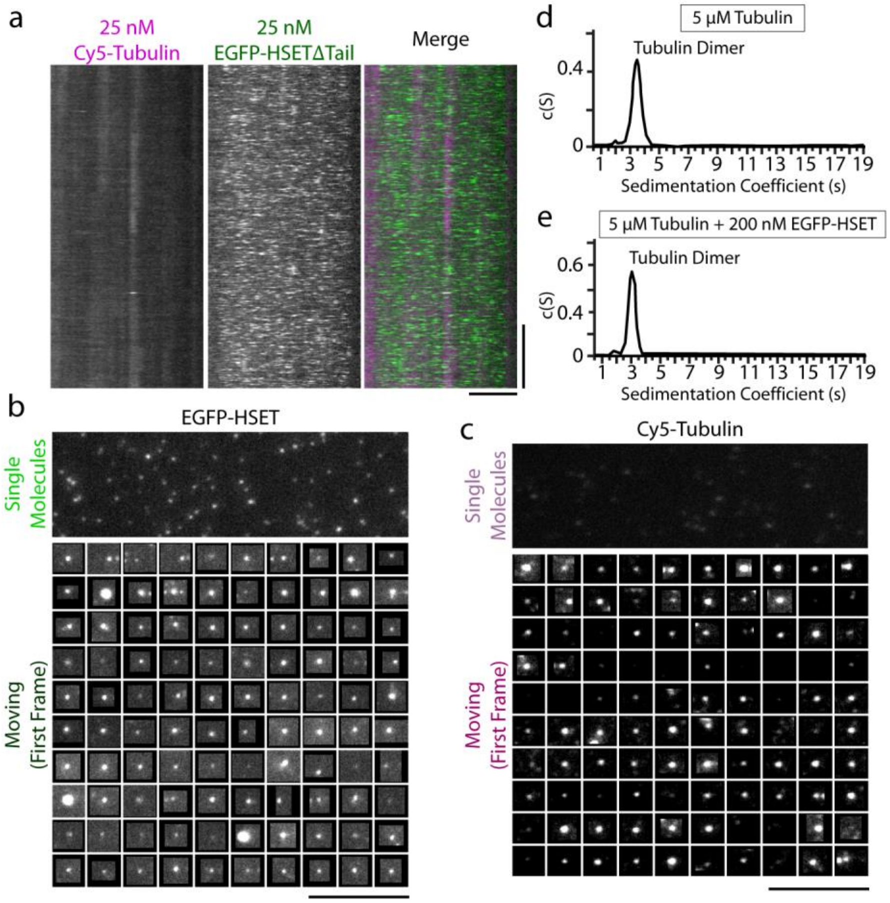

Supplementary Figure 3. Tubulin induces HSET clustering upon binding to tail domain. **(a)** Cy5-tubulin (magenta) and EGFP-HSETΔTail (green) were mixed in BRB80 + 50 mM KCl, observed via two-color time-lapse TIRF, and visualized by kymograph at the indicated concentrations, where EGFP-HSETΔTail showed no observable processive motility (x-scale, distance, 5 μm; y-scale, time, 1 min). **(b)** Representative TIRF images of EGFP-HSET diluted to single-molecule levels and adhered to a glass cover slip (top) compared to the first frame of moving EGFP-HSET (1 nM) after the addition of 20 μM tubulin (bottom, n = 100 particles chosen randomly). Images are processed and acquired identically to illustrate differences in intensity. Data corresponds to Fig. 3b, top. **(c)** Representative TIRF images of Cy5-tubulin diluted to single-molecule levels and adhered to a glass cover slip (top) compared to the first frame of moving Cy5-tubulin (10 nM) in the presence of 10 nM unlabeled HSET with 1 mM GTP present (bottom). Images are processed and acquired identically to illustrate differences in intensity. Data correspond to Fig. 3c. Scale bars, 10 μm. **(d)** The sedimentation velocity profile of 5 μM tubulin as obtained by sedimentation velocity analytical ultracentriguation (AUC) analysis (representative profile). The calculated *s* [c(s)] is plotted *versus* the sedimentation coefficient (S), and the profile fit to a continuous sedimentation distribution. The observed frictional ratio is 1.41 and the r.m.s.d. value is 0.0075. For the prominent peak, S = 3.397, which corresponds to an estimated molecular mass of 102 kDa. **(e)** The sedimentation velocity profile of 5 μM tubulin plus 200 nM EGFP-HSET (representative profile). The observed frictional ratio

is 1.57 and the r.m.s.d. value is 0.0091. For the prominent peak, S = 3.075, which corresponds to an estimated molecular mass of 80 kDa.

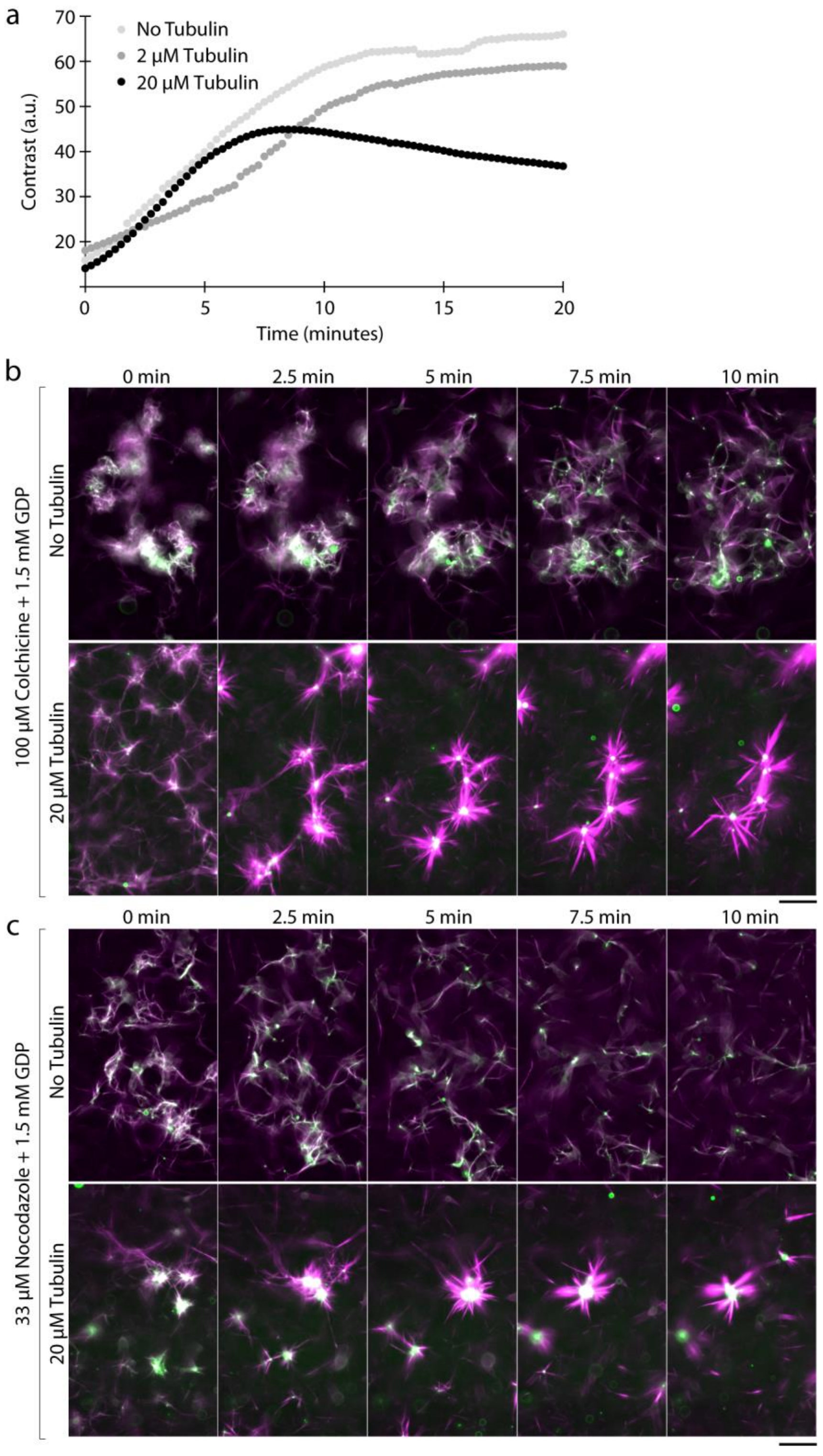

Supplementary Figure 4. Soluble (non-MT) tubulin rescues HSET self-organization independent of polymerization. (a) Representative time course of self-organization by HSET upon tubulin addition (single fields corresponding to Fig. 4a) as monitored by contrast (standard deviation) in the tubulin channel. Unlabeled tubulin concentration is indicated in figure inset. **(b-**

c) EGFP-HSET driven self-organization of GMPCPP-stabilized MTs in the absence of MT polymerization. Experiments were performed identically to (a) but in the absence of taxol and the presence of saturating colchicine/GDP (b) or nocodazole/GDP (c) to prevent polymerization. Technical replicates of experiments in (b-c) were repeated $n \geq 2$ times for each condition, and representative images are shown. Scale bar, 50 μm.

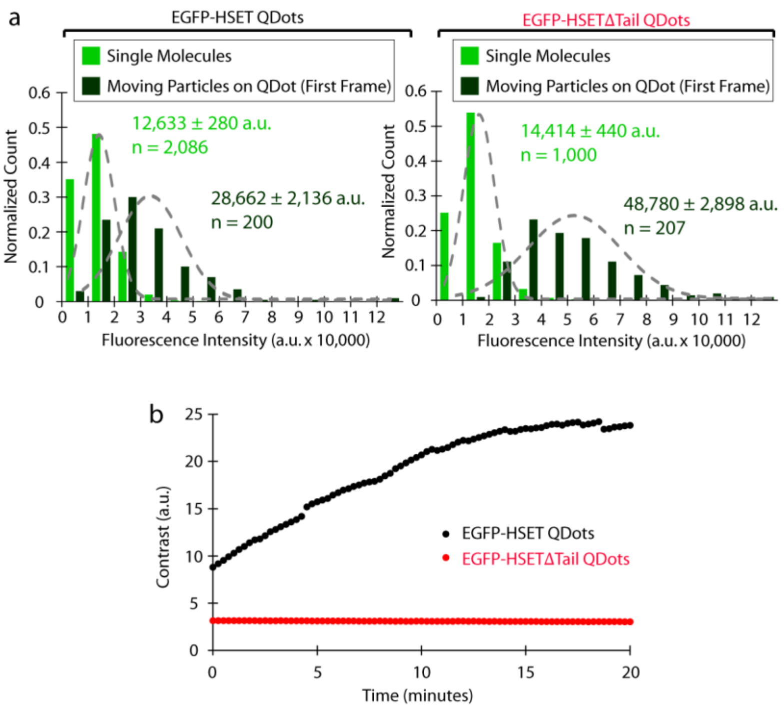

Supplementary Figure 5. Multiple HSET motors conjugated to quantum dots drive aster self-assembly of GMPCPP MTs. (a) Verification of multi-motor assembly of HSET on QDots. After EGFP-HSET (left) or EGFP-HSETΔTail (right) was conjugated to streptavidin QDots via the N-terminal 6x-His tag and biotin-anti-His antibody at a 3:1 ratio (see Fig. 5a), these HSET-QDot complexes were observed by time-lapse TIRF in motility assays. The fluorescence intensity of the first frame of moving EGFP-HSET particles was determined by Gaussian fit, and intensity distributions were plotted as histograms for the population (single EGFP-HSET motors, light green, first frame of moving EGFP-HSET particles, dark green). Data are reported as the mean intensity values (insets) from CDF fitting to normal distributions, ± the 95% CI from bootstrapping from two independent experiments where N ≥ 4 movies for each condition. Note that EGFP-HSET-QDots contain less motors per QDot on average; this is likely due to the large size of the N-terminal tail domain limiting QDot binding. **(b)** Representative time course of self-organization by HSET-QDot assemblies (single fields corresponding to (Fig. 5e)) as monitored by contrast (standard deviation) in the tubulin channel. HSET-QDot assembly type is indicated in figure inset.

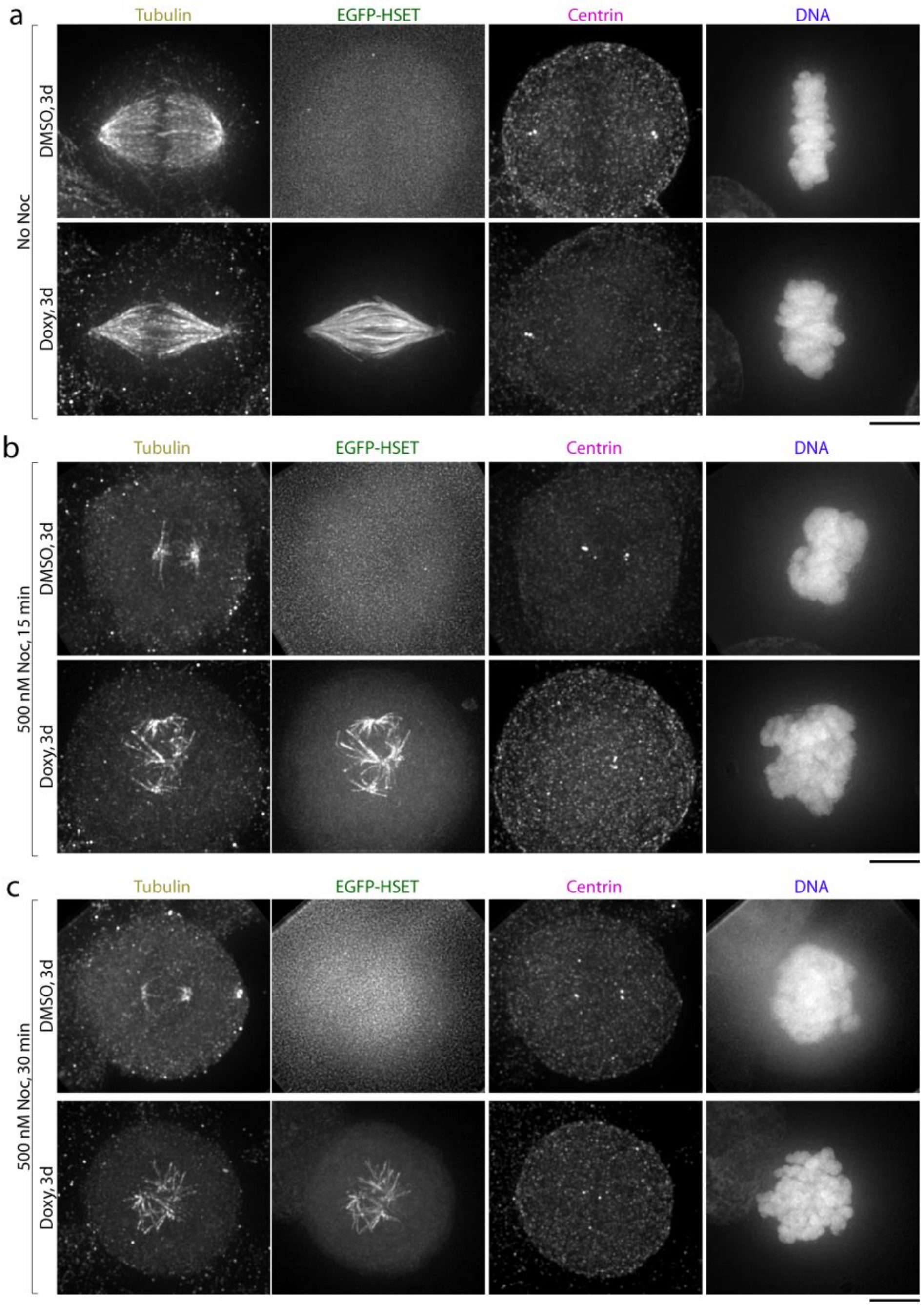

Supplementary Figure 6. Increasing the relative level of soluble (non-MT) tubulin promotes HSET-driven aster formation in fixed cells. (a) Spectrally unmixed images corresponding to Fig. 6b. After pretreating cells for 3d with doxycycline/DMSO, representative z-stacks of metaphase cells where cells were fixed, then (left to right): immunostained for tubulin, EGFP-HSET was imaged directly, immunostained for centrin, and stained for DNA (Hoechst). **(b)** Spectrally unmixed images corresponding to Fig. 6c. After pretreating cells for 3d with doxycycline/DMSO then treating cells for 15 min with 500 nM nocodazole, representative z-stacks of cells where cells were fixed, then (left to right): immunostained for tubulin, EGFP-HSET was imaged directly, immunostained for centrin, and stained for DNA (Hoechst). **(c)** After

pretreating cells for 3d with doxycycline/DMSO then treating cells for 30 min with 500 nM nocodazole, representative z-stacks of cells where cells were fixed, then (left to right): immunostained for tubulin, EGFP-HSET was imaged directly, immunostained for centrin, and stained for DNA (Hoechst). Conditions correspond to Fig. 6d (right), and a representative image is shown. Scale bars, 5 μm.

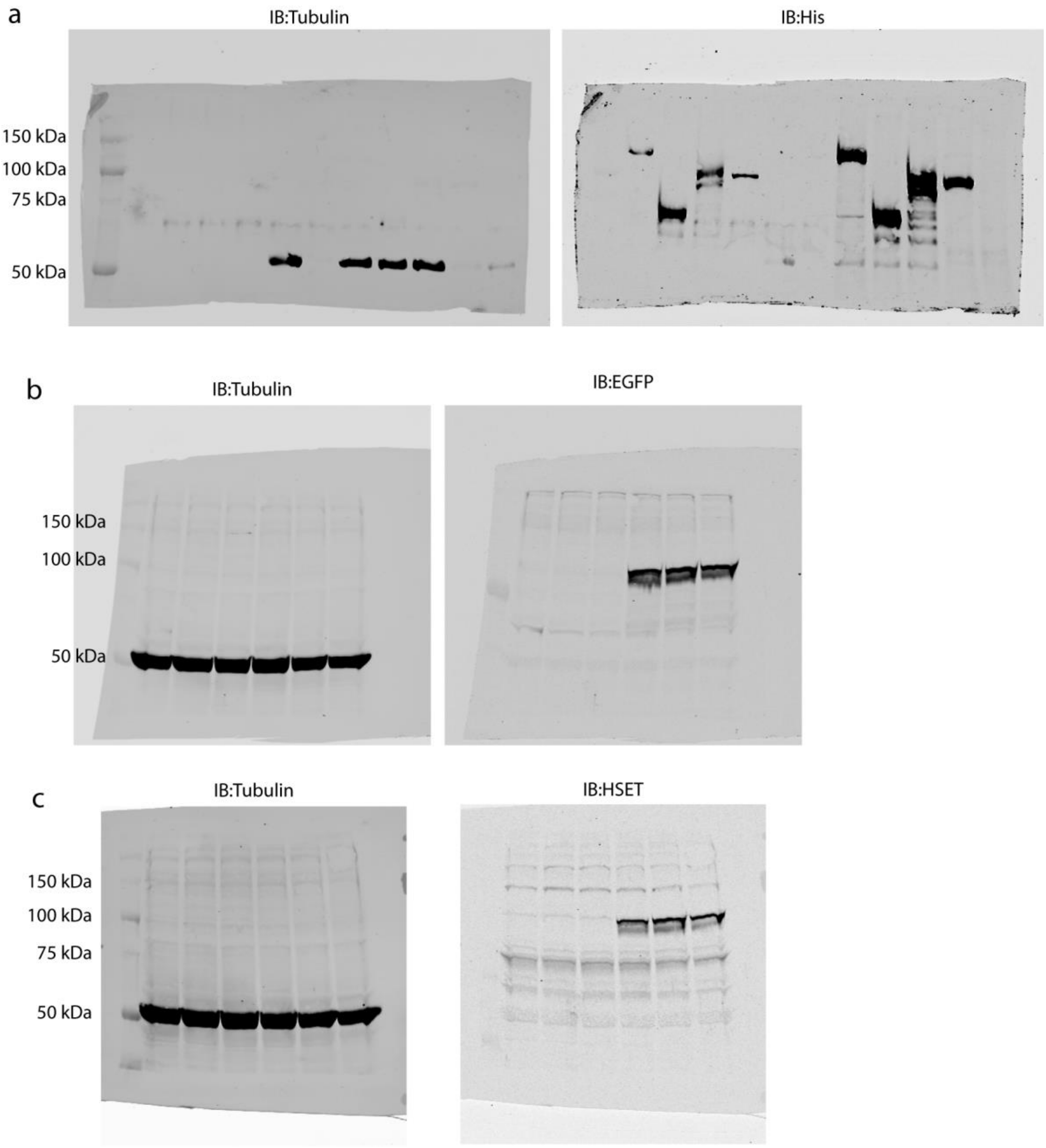

Supplementary Figure 7. Full blots corresponding to portions presented in main paper. (a) Full blots corresponding to Fig. 3a. **(b)** Full blots corresponding to Fig. 6a, top. **(c)** Full blots corresponding to Fig. 6a, bottom. See legends in main text for experimental details.
