# Supplementary material for: Microtubule minus-end aster organization is driven by processive HSET-tubulin clusters
Source: Nat Commun. 2018 Jul 9;9:2659. doi: 10.1038/s41467-018-04991-2 (PMC6037785; doi:10.1038/s41467-018-04991-2)
Supplement: Supplementary file 3 — Description of Additional Supplementary Files [file 41467_2018_4991_MOESM3_ESM.docx]

**Description of Additional Supplementary Files**

File Name: Supplementary Movie 1

Description: Full-length HSET organizes growing MTs into asters. Corresponds to Fig. 1b. Selforganization of growing MTs by HSET. 20 µM tubulin (10% Alexa594-labeled, magenta) was mixed in assay buffer with the indicated EGFP-HSET truncation (green) and monitored by time-lapse microscopy at 37°C. With the exception of EGFP-HSETΔTail (20 nM), all HSET constructs were present at 100 nM. HSET construct, scale bar and time stamp indicated on movie.

File Name: Supplementary Movie 2

Description: GMPCPP-stabilized MTs are not organized into asters in the absence of soluble tubulin. Corresponds to Fig. 1c. Self-organization of GMPCPP-stabilized MTs by HSET. Alexa594-labeled GMPCPP MTs (10% labeled, 1 µM tubulin in polymeric form, magenta) were mixed in assay buffer with the indicated EGFP-HSET truncation (green) and monitored by time-lapse microscopy at 37°C. With the exception of EGFP-HSETΔTail (20 nM), all HSET constructs were present at 100 nM. HSET construct, scale bar and time stamp indicated on movie.

File Name: Supplementary Movie 3

Description: Single HSET motors show bidirectional diffusion rather than processive motion. Corresponds to Fig. 2a-c. EGFP-HSET truncations (green, indicated) were diluted in P12 buffer and monitored by time-lapse TIRF on GMPCPP-stabilized MTs (magenta). The indicated constructs were used at the following concentrations: EGFP-HSET and EGFP-HSETΔMotor, 50 pM. EGFP-HSETΔTail, 250 pM. Scale bar and time stamp indicated on movie.

File Name: Supplementary Movie 4

Description: Tubulin activates HSET processivity on single MTs. Corresponds to Fig. 2d-e. EGFP-HSET in BRB80 + 50 mM KCl was monitored by time-lapse TIRF in the presence (left) or absence (right) of 2 µM soluble tubulin. EGFP-HSET concentration, scale bar, and time stamp are indicated on movie.

File Name: Supplementary Movie 5

Description: Three-color imaging of tubulin transport by EGFP-HSET. Related to Fig. 2g. GMPCPP MTs (red, row 1), 10 nM EGFP-HSET (green, row 2), and 40 nM Cy5-tubulin (magenta, row 3) were observed near-simultaneously by time-lapse TIRF in BRB80 + 50 mM KCl, Row 4 shows merged image. Scale bar and time stamp are indicated on movie.

File Name: Supplementary Movie 6

Description: Soluble tubulin activates MT aster formation of GMPCPP-stabilized MTs. Corresponds to Fig. 4a. Alexa594-labeled GMPCPP MTs (10% labeled, 1 µM tubulin in polymeric form, magenta) were mixed in assay buffer with EGFP-HSET (100 nM, green) and monitored by time-lapse microscopy at 37°C. Unlabeled tubulin was added to the reaction at the indicated concentration (left, 0 µM; middle, 2 µM; right, 20 µM). Scale bar and time stamp are indicated on movie.

File Name: Supplementary Movie 7

Description: Conjugation of multiple HSET motors to QDots activates processive motility. Corresponds to Fig. 5a. EGFP-HSET (top, 1 nM EGFP-HSET : 0.33 nM QDot) or EGFP-HSETΔTail (bottom, 0.5 nM EGFP-HSETΔTail : 0.17 nM QDot) was conjugated to QDots via the N-terminal 6x-His tag at a 3:1 ratio and visualized via TIRF. Green, HSET; Magenta, GMPCPP-MT. Scale bar and time stamp are indicated on movie.

File Name: Supplementary Movie 8

Description: EGFP-HSET on QDots forms MT asters from GMPCPP-stabilized MTs. Corresponds to Fig. 5e. Alexa594-labeled GMPCPP MTs (10% labeled, 1 µM tubulin in polymeric form, magenta) were mixed in assay buffer with the indicated motor-QDot complexes (21:7 nM motor-QDots, green) and monitored by time-lapse microscopy at 37°C. Scale bar and time stamp are indicated on movie.

File Name: Supplementary Movie 9

Description: Formation of asters in EGFP-HSET cells after nocodazole addition. Corresponds to Fig. 6e. EGFP-HSET HeLa cells were treated with doxycycline for 3 days to induce maximal expression, and 500 nM nocodazole was added to increase relative levels of soluble tubulin. Maximum intensity z-projections from the EGFP-HSET channel are shown at 3 min intervals. Scale bar and time stamp are indicated on the movie.
